# The role of vitreous management in the posterior capsule rupture associated with cataract surgery on the protection of the corneal endothelium

**DOI:** 10.22336/rjo.2022.26

**Published:** 2022

**Authors:** Adina-Iuliana Milcu, Sînziana Luminița Istrate, Emil Ungureanu, Călin Petru Tătaru, Mihnea Munteanu, Ovidiu Borugă, Mădălina-Casiana Palfi (Salavat), Andrei Anghel

**Affiliations:** * Department of Ophthalmology, “Victor Babeș” University of Medicine and Pharmacy, Timișoara, Romania; ** Department of Ophthalmology, “Carol Davila” University of Medicine and Pharmacy, Bucharest, Romania; *** Department of Biochemistry, “Victor Babeș” University of Medicine and Pharmacy, Timișoara, Romania

**Keywords:** posterior capsule rupture, vitrectomy, corneal endothelium

## Abstract

**Introduction**:

Advances in technology and technique have led to a significant improvement in the prognosis after cataract surgery. However, there are complications that can significantly affect this prognosis, such as posterior capsule rupture and corneal decompensation. For vitreous prolapse associated with posterior capsule rupture, classic or pars plana anterior vitrectomy is required.

**Aim**:

The aim of the study was to compare corneal endothelial cell destruction after cataract surgery associated with posterior capsule rupture and classical and pars plana anterior vitrectomy, respectively.

**Material and method**:

The study was prospective, on 12 consecutive cases of cataract surgery associated with posterior capsule rupture. Classical anterior vitrectomy was performed in group A, with 5 patients, while pars plana anterior vitrectomy was performed in group B. For all cases, the Stellaris phacoemulsification device (Baush & Lomb, tm) and the associated vitrectomy device was used.

**Results**:

Pars plana anterior vitrectomy had a statistically significant lower rate of corneal endothelial damage, both in absolute value and as a percentage of initial density.

**Conclusions**:

Pars plana anterior surgery is a somewhat unfamiliar technique for anterior pole surgeons. But it is easy to learn and brings a decrease in the rate of damage to the corneal endothelium.

## Introduction

The prognosis after cataract surgery has seen dramatic advances associated with advances in technology, surgical techniques, and increasingly early stages surgery. However, there are still various complications associated with cataract surgery and the most common complication encountered is posterior capsule rupture.

Anterior vitrectomy was first described by David Kasner who, in 1961, performed open-sky subtotal vitrectomy on a post-traumatic eye, then on two patients with amyloidosis in 1966-1967. In 1971, Cerasoli and Kasner published the first significant paper on anterior vitrectomy [**1**], performed by scissor sectioning the exposed vitreous at the level of the wound using cellulose sponges. However, this technique is associated with significant vitreoretinal traction and possible subsequent complications.

With the advent of vitrectomies associated with phacoemulsification devices, anterior vitrectomy has become much safer and, when necessary, can and should be performed by the anterior pole surgeon. However, this surgery is performed near the base of the vitreous and is not necessarily simple. When a posterior capsule rupture occurs associated with cataract surgery, it is mandatory to restore the anterior chamber, preferably with dispersive viscoelastic, before removal of phacoemulsification specimen. However, if vitreous prolapse still occurs, vitrectomy will be required. There are two variants that can be used in the management of the posterior capsule rupture associated with vitreous prolapse, namely classical anterior vitrectomy using corneal incisions or pars plana vitrectomy.

*Classic anterior vitrectomy* is done using counter-incisions, never the main incision. The use of infusion is recommended, while dry vitrectomy may be associated with hypotonia, myosis, and suprachoroidal hemorrhage. The infusion should use a different counter-incision than vitrectomy and a higher cutting rate is recommended to minimize vitreoretinal traction [**2**].

*Pars plana vitrectomy* is performed by using an incision of about 3.5 mm from the limbus, usually with the 23 G vitrectomy, with or without trocar. The infusion can be performed through a corneal counter-incision. The technique can also be used in cases with topical or intracameral anesthesia [**3**].

Cataract surgery is invariably associated with a decrease in corneal endothelial cell density. This decrease is significantly greater in surgery associated with vitrectomy and may be a significant risk factor for visual prognosis [**4**,**5**].

## Aim

The aim of the study was to compare corneal endothelial cell destruction after cataract surgery associated with posterior capsule rupture and classical and pars plana anterior vitrectomy, respectively.

## Material and methods

The study was prospective, on 12 consecutive cases of cataract surgery associated with posterior capsule rupture and was conducted between 2019 and 2021. While the initial procedure, until the posterior capsule rupture occurred, was performed by different surgeons, the subsequent resolution of the case, including associated vitrectomy, was performed by the same surgeon with the same phacoemulsification device and associated vitrectomy. In all cases, a lens was implanted in the sulcus.

Inclusion criteria:

- patients with cataract surgery in whom posterior capsule rupture with vitreous prolapse occurred and anterior vitrectomy was required.

Exclusion criteria:

- patients with cataract surgery and posterior capsule rupture in whom there was a posterior dislocation of lens material and who required posterior vitrectomy, which was performed later on;

- patients with a history of previous concussion ocular trauma or ocular plaques.

Patients were divided into two groups:

Group A, consisting of 5 patients, in whom classical anterior vitrectomy was performed.

**Table 1:** Patients with classical anterior vitrectomy performed

	Cataract Type	Phacoemulsification Time (EPT) (seconds)	Preoperative endothelial cell density (/ mm2)	Density of endothelial cells at 3 months postoperatively (/ mm2)
B.I., 76-year-old, F	3+	7.9	2028	1768
A.J., 84-year-old, M	2-3+	4.6	1954	1712
S.C., 62- year-old, F	4+	12.5	2330	2016
M.P., 81-year-old, F	3-4+	8.5	2596	2133
S.E., 86-year-old, F	3+	5.8	2154	1867

Group B, consisting of 7 patients, in whom a pars plana anterior vitrectomy was performed.

**Table 2:** Patients with pars plana anterior vitrectomy performed

	Cataract Type	Phacoemulsification Time (EPT) (seconds)	Preoperative endothelial cell density (/ mm2)	Density of endothelial cells at 3 months postoperatively (/ mm2)
A.B. 69-year-old, M	4+	14.4	2436	2284
C.F., 76-year-old, M	3+	6.6	1954	1782
D.H., 82-year-old, F	2+	3.5	2208	2068
D.S., 91-year-old, F	3+	9.2	2312	2081
N.A., 78-year-old, F	3-4+	5.7	2644	2463
B.N., 83-year-old, F	2-3+	6.3	2251	2053
R.V., 72-year-old, M	3+	7.4	1754	1496

## Results

There was no statistically significant difference (p = 0.44572) between Group A (7.5857s ± 3.2067) and Group B (7.86s ± 3.7716) in terms of phacoemulsification time. Also, there was no statistically significant difference (p = 0.476669) between the number of preoperative endothelial cell counts, which were 2212.4 ± 320.1006/ mm2 for Group A and 2222.7143 ± 273.6211/ mm2 for Group B.

In terms of endothelial cell counts at 3 months postoperatively, the difference was not statistically significant (p = 0.207606), probably due to a small group. However, this aspect is not the most relevant. Even though the groups were similar intraoperatively, they were not completely identical. We considered as more relevant the decrease in endothelial cell counts in absolute value and, respectively, in percentage compared to the initial examination.

The absolute decrease in endothelial cell count was statistically significantly (p = 0.0038735) higher in Group A (314.8 ± 108.0752/ mm2) than in Group B (190.2857 ± 89.1369/ mm2). The decrease in endothelial cell density, as a percentage compared to preoperative examination, was statistically significantly different (p = 0.0037455), with a smaller decrease in Group B (8.8129 ± 2.745%) compared to Group A (14.042 ± 2.6847%).

## Discussions

Patients have very high expectations for the outcome of cataract surgery. This often-routine surgery can however be complicated by a posterior capsule rupture, which must therefore be dealt with as efficiently as possible.

When capsular rupture occurs, viscoelastic injection through a counter-incision before withdrawing the phacoemulsification sample is essential. Repeated injection of viscoelastic ± scaffolding-type pseudophakia implantation techniques is necessary to prevent and minimize the vitreous prolapse.

The cystoid macular edema that is more commonly associated with these procedures is never caused by vitrectomy itself, but by iris trauma associated with retractors and surgical manipulation.

## Conclusions

The main limitation of this study is the small number of patients enrolled. Even so, pars plana anterior vitrectomy was statistically significantly more effective in protecting the corneal endothelium. If for routine cases, with good corneal endothelial cell density, it does not necessarily bring a significant additional benefit; for cases in which the endothelium is damaged it can represent the difference between a good and a significantly worse visual prognosis.

Pars plana anterior surgery is a somewhat unfamiliar technique for anterior pole surgeons. However, it is easy to learn and brings a decrease in the rate of corneal endothelial damage.

## References

[1] Cerasoli JR, Kasner D (1971). A follow-up study of vitreous loss during cataract surgery managed by anterior vitrectomy. Am J Ophthalmol.

[2] Nanavaty MA, Ashena Z (2021). Face down’ anterior vitrectomy for unexpected posterior capsule rupture as an alternative to pars plana vitrectomy. Eye.

[3] Thornton IL, McMains BK, Snyder ME (2018). Long-term safety and efficacy of single-port pars plana anterior vitrectomy with limbal infusion during anterior segment surgery. J Cataract Refract Surg.

[4] Nanu RV, Ungureanu E, Istrate SL, Vrapciu A, Cozubas R, Carstocea L, Voinea LM, Ciuluvica R (2018). Investigation of importance of the structural parameters of the eyeball and of the technical parameters of cataract surgery on corneal endothelial changes. Rom J Ophthalmol.

[5] Ho JW, Afshari NA (2015). Advances in cataract surgery: Preserving the corneal endothelium. Curr Opin Ophthalmol.

